# Confined toluene within InOF-1: CO_2_ capture enhancement†

**DOI:** 10.1039/c9ra05991a

**Published:** 2019-10-15

**Authors:** L. Pamela Garrido-Olvera, Jonathan E. Sanchez-Bautista, Daniel Alvarado-Alvarado, Bruno Landeros-Rivera, J. Raziel Álvarez, Rubicelia Vargas, Eduardo González-Zamora, Jorge Balmaseda, Hugo A. Lara-García, Ana Martínez, Ilich A. Ibarra

**Affiliations:** Laboratorio de Fisicoquímica y Reactividad de Superficies (LaFReS), Instituto de Investigaciones en Materiales, Universidad Nacional Autónoma de México Circuito Exterior s/n, CU, Coyoacán 04510 Ciudad de México Mexico argel@unam.mx; Departamento de Química, Universidad Autónoma Metropolitana-Iztapalapa San Rafael Atlixco 186, Col. Vicentina, Iztapalapa C. P. 09340 Ciudad de México Mexico brunolanderos@hotmail.com ruvf@xanum.uam.mx; Instituto de Investigaciones en Materiales, Universidad Nacional Autónoma de México Circuito Exterior S/N, CU, Coyoacán 04510 Ciudad de México Mexico; Instituto de Física, Universidad Nacional Autónoma de México Circuito de la Investigación científica S/N, CU, Del. Coyoacán 04510 Ciudad de México Mexico

## Abstract

The toluene adsorption properties of InOF-1 are studied along with the confinement of small amounts of this non-polar molecule revealing a 1.38-fold increase in CO_2_ capture, from 5.26 wt% under anhydrous conditions to 7.28 wt% with a 1.5 wt% of pre-confined toluene at 298 K. The InOF-1 affinity towards toluene was experimentally quantified by Δ*H*_ads_ (−46.81 kJ mol^−1^). InOF-1 is shown to be a promising material for CO_2_ capture under industrial conditions. Computational calculations (DFT and QTAIM) and DRIFTs *in situ* experiments provided a possible explanation for the experimental CO_2_ capture enhancement by showing how the toluene molecule is confined within InOF-1, which constructed a “bottleneck effect”.

## Abstract 2

The confinement of small amounts of toluene demonstrated an enhanced CO_2_ capture for InOF-1 as a result of a bottleneck effect and synergistic interactions.

## Graphical abstract

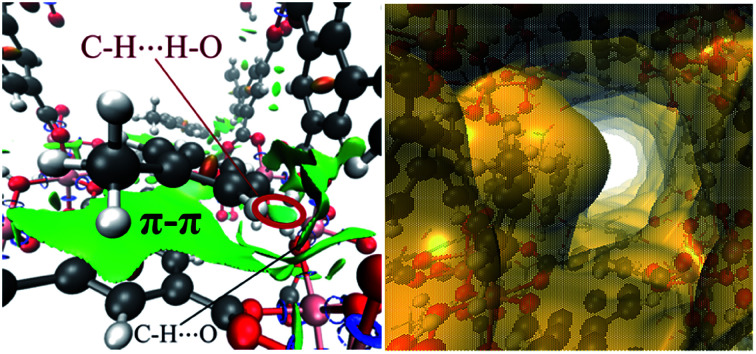

## Introduction

In the last decades, global warming has become one of the biggest threats that our civilization faces. Scientists agree that this phenomenon is related to the cumulative emissions in the atmosphere of carbon dioxide (CO_2_) and the increase of anthropogenic activities which generate the so-called greenhouse gases (GHG).^1^ According to the International Energy Agency (IEA)^2^ the energy industry is the sector with the highest CO_2_ emissions, resulting in 42% of the global total emissions in 2016. In spite of the current necessity for using renewable energy sources, and different efforts to develop new technologies, the production of energy from fossil fuel sources will, unfortunately, remain dominant for the coming years.

Undeniably, identifying present problems and working on multiple solutions to reduce the atmospheric CO_2_ levels is critical to mitigate the environmental threats and problems that global warming present. Sequestration of CO_2_ represents a promising method for mitigation, and a key step solution to effectively reduce the CO_2_ levels in ecosystems. However, in order to solve the problem of global warming and high concentration levels of CO_2_, the search and development for a cost-effective capture technology is needed. Several technologies and materials have been proposed to mitigate the emissions, for instance, the carbon sequestration of biomass feedstock, microalgae.^3^ On the other hand, Carbon Capture and Storage (CCS) are a wide group of technologies being developed in aim to capture and safely storage the CO_2_ emitted from industrial processes before it enters the atmosphere.^4,5^

Unfortunately, CO_2_ is not the only GHG which negatively contributes to affect the environment, but there is a vast list of chemical pollutants that are quite abundant in both the outdoor environment and indoor air quality.^6^ Volatile organic compounds (VOCs), and more specifically BTEX (benzene, toluene, ethylbenzene and xylene) are key materials in the organic chemistry industry. Organic chemicals are used in household products, which can release organic compounds when using them.^7^ Recent studies indicate that VOCs are considered a major group of air pollutants and a serious concern due to their major environmental and human health hazard.^8^

Multiple methods of gas capture materials are used at present for the sequestration for both CO_2_ and VOCs, from activated carbon to zeolites, calcium oxides, hydrotalcites, lithium zirconate, and metal–organic frameworks (MOFs).^9^

MOFs emerged as a promising alternative to solve various problems and to overcome limitations experienced with solid sorptive materials. The structure of MOFs consists of metal centers connected by organic ligands which results in a porous material with high surface area, diverse size of the cavities and thus, different applications have been found.^10^ Depending on the selection of the organic ligand and the metal centre, the resulting MOF material will have distinctive properties. As a result of this flexibility, MOFs sorption selectivity towards CO_2_ (in comparison to other gases *e.g.*, CH_4_, N_2_ and H_2_), makes them one of the principal candidates for capture and separation of CO_2_.

Recent studies have demonstrated that the inclusion of different molecules, within nanometre scale materials induce an increase the gas solubility in which Henry's law might be limited.^11^ This phenomenon is also known as “gas-over solubility” and can modify the physicochemical properties of the confined molecules (typically solvents).^12,13^ Several research groups have reported an increase of gas solubility when relatively large amounts of molecules are pre-adsorbed in mesoporous solid materials. Luzar and Bratko predicted (Monte Carlo calculations) an increase on the solubility of different gases (O_2_, N_2_, CO_2_, and Ar) when water molecules are included in a hydrophobic environment.^14,15^ Pera-Titus *et al.*, reported an enhancement on the H_2_ solubility when different molecules (*i.e.*, CS_2_, CHCl_3_, CCl_4_, *n*-C_6_H_14_, H_2_O, and EtOH) were confined.^13,16^ In more recent studies it has been demonstrated that, in water stable MOFs, small amounts of pre-absorbed water (confined) within their pores, it can considerably enhance CO_2_ capture.^17^ Experimental data in different water stable MOFs such as InOF-1,^18^ NOTT-400,^19^ and Mg-CUK-1^20^ allowed to elucidate the role of confined water molecules within these MOFs, and how the interactions (hydrogen binding) of water with the hydroxo functional groups (μ_2_-OH) are the key for the CO_2_ capture enhancement.^17^

Additionally, MOFs exhibit high capacity and selectivity for VOCs, as well as promising sorption and separation capacities.^21^ Regardless of the characteristics of some previously studied MOFs, their application as sorptive materials for the removal of VOCs (such as toluene) has been a challenging task. A vast group of capture methods have been investigated such as condensation, catalytic oxidation, biodegradation, and adsorption methods.^22^

InOF-1 is a water-stable microporous MOF material, which is constructed from a flexible BPTC (biphenyl-3,3′,5,5′-tetracarboxylate) and based on a binuclear [In_2_(μ_2_-OH)] building block.^23^ Sequestration of CO_2_ in InOF-1 has previously been demonstrated under anhydrous conditions and with the pre-adsorption (confinement of small amounts) of different polar molecules,^24–28^ resulting in the increase on the total CO_2_ capture. In all these examples the hydrogen bond between these polar molecules and the hydroxo functional group, showed to be the dominant intermolecular interaction to enhance the CO_2_ uptake. In this study we present the capture of CO_2_ with a pre-confined solvent that happens not to be a hydrogen bonding donator within the InOF-1 material: toluene. The CO_2_ capture properties of InOF-1 and the adsorption of toluene were experimentally studied and correlated with computational calculations.

## Experimental details

### Chemicals

Indium nitrate (In(NO_3_)_3_), biphenyl-3,3′,5,5′-tetra-carboxylic acid (H_4_BPTC), *N*,*N′*-dimethyl formamide (DMF), acetonitrile (CH_3_CN), and nitric acid (HNO_3_) were obtained from Sigma-Aldrich and used as received without any further purification. Water (H_2_O) and acetone (Me_2_CO), used for washing purposes, were technical grade.

### Synthesis and activation

InOF-1 = [In_2_(OH)_2_(BPTC)] = In_2_(OH)2(biphenyl-3,3′,5,5′-tetra-carboxylate), was synthesised according to the previously reported procedure methodology by Hong *et al.*^23^ The precipitate is formed by dissolving In(NO_3_)_3_·5H_2_O (156 mg, 0.4 mmol), and H_4_BPTC (33 mg, 0.10 mmol) in DMF (5 mL), MeCN (5 mL), and HNO_3_ (0.2 mL, 65 wt%), in a sealed pressure tube. The clear solution is heated at a temperature of 85 °C (358 K) in an oil bath for 72 h. The pressure tube was cooled down to room temperature over a period of 12 h and the colourless crystalline product was separated by filtration, washed with DMF (5 mL), and finally dried in air leading to a yield of 73% (based on ligand). Powder X-ray diffraction (PXRD) analysis was performed on the InOF-1 sample to confirm the MOF structure and control the purity of the synthesised material. The as-synthesised sample was acetone-exchanged and then activated at 453 K (810 °C) for 2 h with a constant flow of dry N_2_ gas.

### PXRD experiments

Powder X-ray diffraction (PXRD) analyses were performed in a Bruker AXS D8 Advance system (Cu Kα radiation) to confirm the purity of the synthesised material (see Fig. S1, ESI†). PXRD patterns were recorded under ambient conditions from 5 to 55° (2*θ*) with a 0.02° per step, at a scan rate of 0.8° min^−1^. These patterns were obtained from the as-synthesised material and after the CO_2_ capture experiments.

### Adsorption isotherms for toluene

To record the toluene sorption isotherms, ultrapure grade (99.9995%) N_2_ gas purchased from Praxair was used as a vapour carrier in a DVS Advantage 1 instrument, from Surface Measurement Systems (mass sensitivity: 0.1 μg; RH accuracy: 0.5% RH, vapor pressure accuracy: 0.7% *P*/*P*_0_). Toluene adsorption–desorption isotherms were collected at 308 and 298 K. InOF-1 samples were activated at 453 K for 2 hours under a dry N_2_ flow on the DVS prior to toluene adsorption experiments. In order to obtain the isotherms, a partial pressure method was carried out with controlled changes of partial pressure and a constant temperature in the sample chamber.

### Kinetic CO_2_ uptake experiments

CO_2_ uptake experiments were performed in the same instrument from Surface Measurement Systems (*vide supra*) at constant temperature and CO_2_ flow. Prior to any CO_2_ uptake experiments, InOF-1 samples were fully activated by increasing the temperature to 453 K under a dry N_2_ gas flow for 2 h in the DVS Advantage 1 instrument. A selected partial pressure of 1.5% *P*/*P*_0_ was used to achieve the desired weight percentage of confined toluene within the activated sample of InOF-1. Then, CO_2_ uptake experiments were carried out at 298 K allowing a constant CO_2_ gas flow (100 cm^3^ min^−1^) inside the sample chamber until the sample mass remained constant.

### Computational details

Density Functional Theory (DFT) periodical calculations of the toluene adsorption process, in InOF-1, were performed at B3LYP-D*/POB-TVPZ^29,30^ level of theory employing the Crystal14 software.^31^ The initial structure for the geometry optimisation present the toluene ring laying parallel over one of the biphenyl rings (ligand) of InOF-1. Toluene molecule was fully geometry optimised whilst the rest of the atomic positions and the cell parameters remained fixed. The intermolecular interactions were analysed by means of the quantum theory of atoms in molecules^32^ (QTAIM), the Non-Covalent Interaction index^33^ (NCI) and the electrostatic potential. The QTAIM analysis, as well as void volume computations based on an electron density isosurfaces, was conducted with a program developed by our research group that is in a beta version of GPUAM.^34^ The NCI analysis was performed over a pro-molecular density with the NCIPLOT program.^35^ Once the geometry of the toluene molecule was optimised, a CO_2_ molecule was placed over its aromatic ring and its positions were relaxed, keeping the rest of the system fixed. This computation was carried out using the same procedure stated above.

### DRIFT spectroscopy

Diffuse Reflectance Infrared Fourier Transform (DRIFT) spectroscopy experiments were performed using an environmentally controlled PIKE DRIFT cell with SeZn windows coupled to a Thermo Scientific Nicolet iS50 spectrometer with a DTGS detector. Absorbance spectra were obtained by collecting 64 scans at 4 cm^−1^ resolution. A sample of 25 mg was pre-treated *in situ* under a helium flow of 30 mL min^−1^ at 180 °C for 1 h. After this treatment, the sample was functionalised with toluene (toluene@InOF-1). CO_2_ adsorption was studied using a flow of 30 mL min^−1^ of CO_2_ (5% CO_2_ in Ar). Spectra of the toluene-functionalised InOF-1 were collected at different times.

## Results and discussion

### Toluene sorption studies

**Fig. 1:**
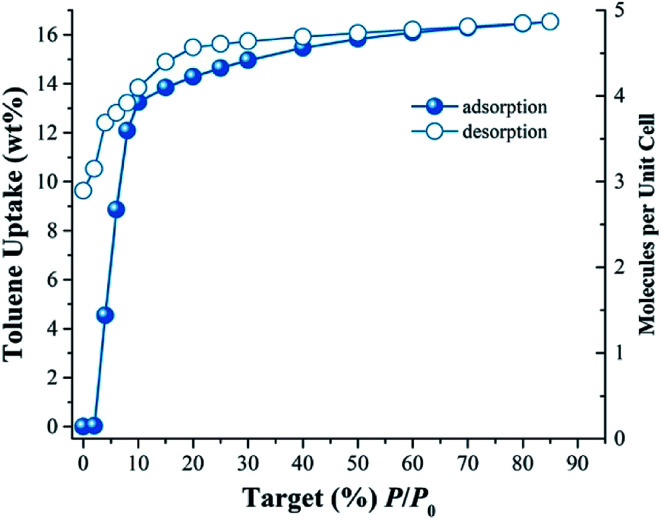
Toluene adsorption–desorption isotherm at 298 K of InOF-1 from 0 to 85 %*P*/*P*_0_.

Fig. 1 shows the toluene adsorption–desorption isotherm for InOF-1 carried out from % *P*/*P*_0_ = 0 to 85 at 298 K. Due to possible favourable host–guest interactions, the uptake rapidly rises with *P*/*P*_0_ (see Fig. 1). The toluene capture quickly increased attaining an uptake of approximately 13.9 wt% (1.5 mmol g^−1^) from 0 to 15% *P*/*P*_0_. The toluene uptake was clearly slower from 15 to 85% *P*/*P*_0_, with a maximum capture of approximately 16.5 wt% (1.8 mmol g^−1^). The desorption phase shows hysteresis more evidently at the low pressures ranges from 0 to 10% *P*/*P*_0_ (see Fig. 1). Thus, a high affinity of InOF-1 towards toluene is shown in the desorption phase. Once desorption is completed, a considerable amount of toluene remained trapped inside the pores of the material (approximately 9.6 wt%, 1.05 mmol g^−1^), which suggests a relatively strong host–guest interaction between toluene and InOF-1.

In order to estimate the isosteric heat of adsorption (Δ*H*_ads_) and to investigate the adsorbate–adsorbent interactions (see Fig S2, ESI†) another toluene adsorption isotherm was carried out at 308 K. The isosteric heat of adsorption was calculated by fitting both adsorption toluene isotherms (298 and 308 K) to the Clausius–Clapeyron equation (see Fig. S3, ESI†), resulting in a Δ*H*_ads_ = −46.81 kJ mol^−1^. The Δ*H*_ads_ value was higher than the molar enthalpy of vaporisation for toluene (Δ*H*_vap_ = 38.01 kJ mol^−1^), which is consistent with previously reported ^25,26,28^values for other confined solvents in the same material, InOF-1 and within the range of previously reported MOFs with toluene sorptive capability.^36^

### CO_2_ capture studies

**Fig. 2:**
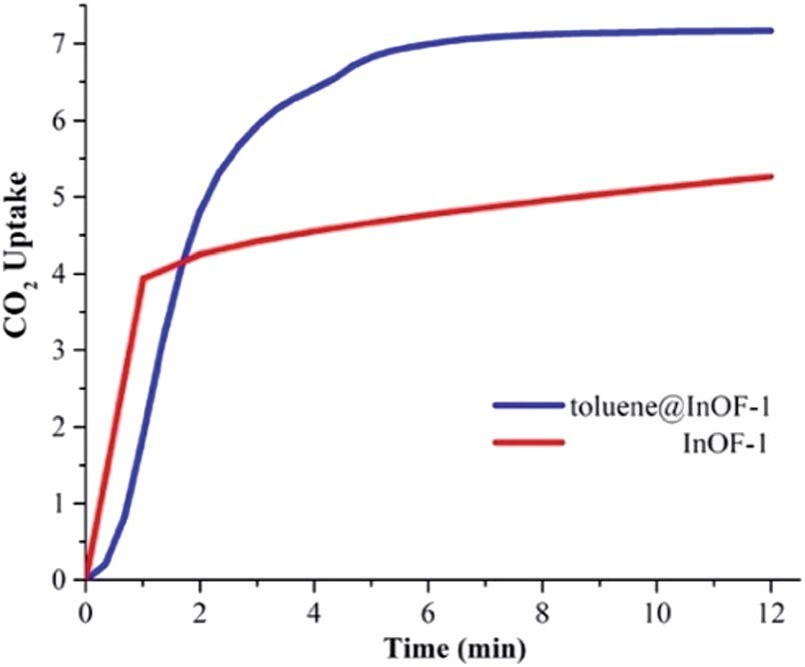
CO_2_ dynamic adsorption experiments performed at 298 K under for InOF-1 (red curve) and toluene@InOF-1 (blue curve); CO_2_ error ±0.05 wt%.

Under anhydrous conditions and at 298 K, the maximum CO_2_ capture was estimated to be 5.26 wt%. This value is in agreement with previously reported CO_2_ captures at the same temperature.^27^ As shown in Fig. 2, after 12 min the mass gain remained essentially constant until the end of the experiment (at approximately 50 min). This means that the CO_2_ capture reached stability after approximately 12 min.

Intrigued by the high affinity of InOF-1 towards toluene (non-polar molecule) we decided to analyse the effect of pre-confining small amounts of toluene and investigate the effect on the CO_2_ adsorption properties of the hybrid material. Since toluene is a non-polar molecule, the formation of hydrogen bonds with the hydroxo functional group (μ_2_-OH) should be less possible. Therefore, the CO_2_ uptake should be approximately the same as under anhydrous conditions.

To verify this hypothesis, we decided to confine (pre-adsorb) a small amount of toluene (1.5 wt%) into the pores of InOF-1. In a DVS Advantage 1 instrument (SMS) with an isothermal and dynamic method, a partial pressure of toluene was selected (1 %*P*/*P*_0_) allowing the pre-adsorption of the non-polar molecule (with dry N_2_ acting as a carrier gas) within the pores of InOF-1. The mass increased to the expected weight percentage value (see Fig. 1), and when the toluene uptake remained constant at 1.5 wt%, the partial pressure was returned to 0% *P*/*P*_0_ maintaining the sample mass constant pre-adsorbing toluene within the pores of InOF-1. This sample was labelled as toluene@InOF-1. Then, a CO_2_ flow (100 cm^3^ min^−1^) was started and allowed into the sample chamber of the DVS instrument. The CO_2_ uptake for toluene@InOF-1 rapidly increased reaching a steady state after only 8 min and fully stabilised (plateau) at 10 min approximately. The maximum amount of CO_2_ captured for toluene@InOF-1 was equal to 7.28 wt% (see Fig. 2). This CO_2_ kinetic-uptake experiment demonstrated that the pre-confinement of 1.5 wt% of toluene within InOF-1 (toluene@InOF-1) resulted in a 1.38-fold CO_2_ increase capture (from 5.26 wt% to 7.28 wt%).

**Fig. 3:**
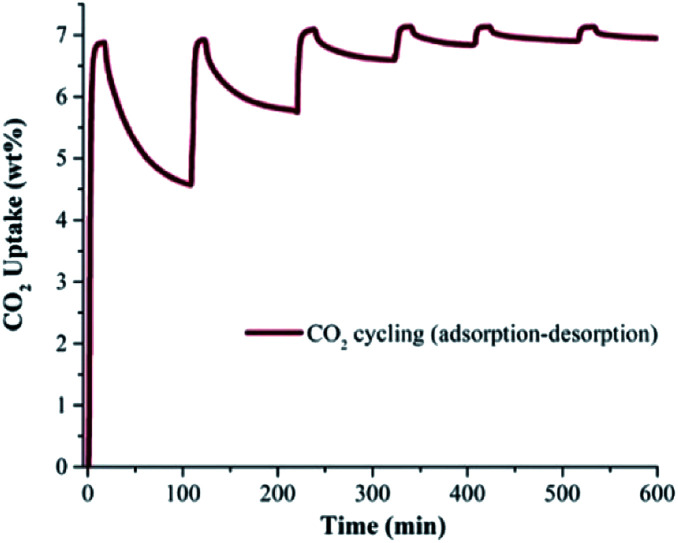
Adsorption–desorption CO_2_ cycling for toluene@InOF-1.

In order to corroborate the CO_2_ capture cyclability of toluene@InOF-1 (evaluation of the regeneration properties and the CO_2_ desorption process), six CO_2_ adsorption–desorption cycles were performed at 298 K. For this, isothermal and dynamic experiments at 298 K were performed in a DVS Advantage 1 instrument (SMS) without the use of any dry N_2_ or other gas as a purge. As shown in Fig. 3, each cycle involved an approximate 20 min adsorption step of CO_2_ followed by an ∼70 min CO_2_ desorption step, to complete a 600 min cycling (adsorption–desorption). By only turning off the CO_2_ flow (absence of a purge gas in the desorption step), only a partial regeneration of toluene@InOF-1 was achieved. Fig. 3 demonstrates that on each cycle the CO_2_ desorption step is reduced even more, with a total CO_2_ capture of 7.20 wt% in the first cycle and a desorption of 4.57 wt%. After six cycles the CO_2_ capture was equal to 7.28 wt% and a desorption of only 0.33 wt%. Remarkably, the capture capacity seems not to be affected by the number of cycles maintaining the maximum enhancement of CO_2_ of 7.28 wt%. This result is relevant since it shows not only an unexpected CO_2_ capture enhancement by the confinement of toluene, but also the retention of the CO_2_ cyclability of toluene@InOF-1 due to an extraordinary high affinity of InOF-1 towards toluene. In addition, it was corroborated by a PXRD experiment, the structure stability of the material after the CO_2_ adsorption–desorption cycling experiments (see Fig. S1, ESI†).

### Computational studies

**Fig. 4:**
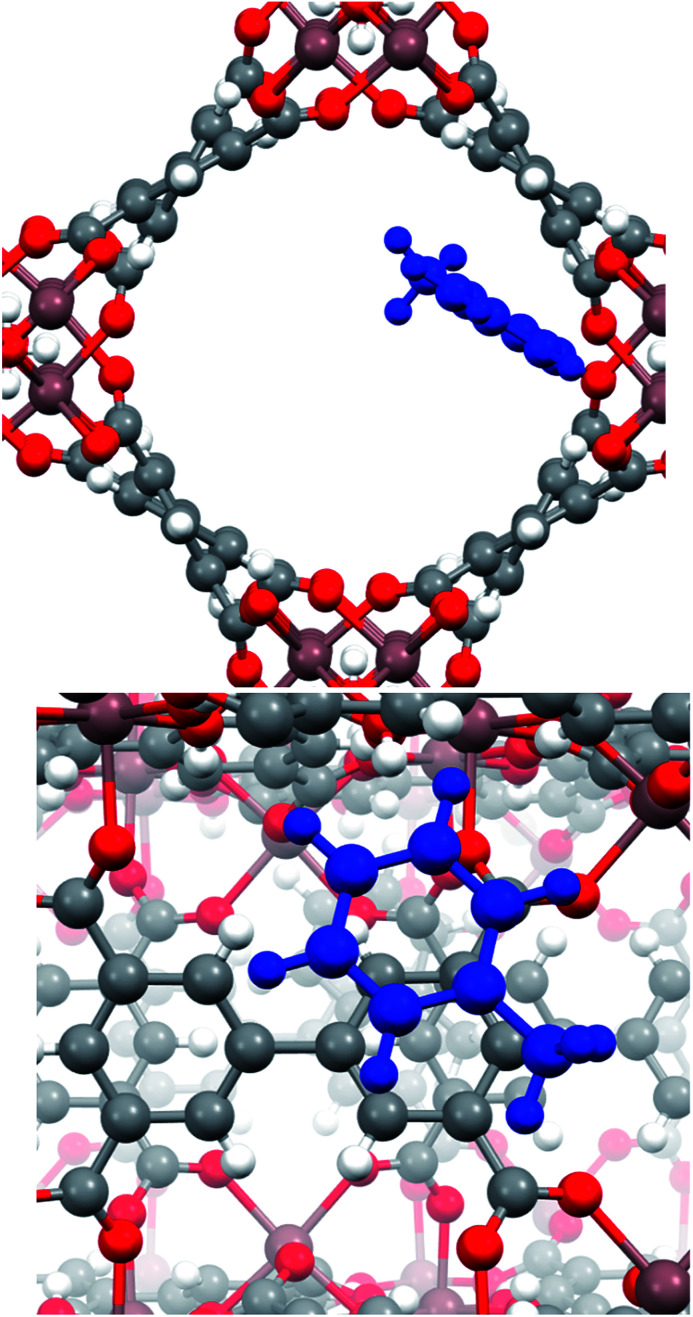
Pore channel (top) and close up (bottom) views of the optimized structure of the toluene molecule (shown in blue) adsorbed within InOF-1 in a parallel displaced geometry. Pink, red, grey and white colours represent indium, oxygen, carbon and hydrogen atoms, correspondingly. This figure was generated with the Mercury software.^40,41^

In order to investigate more about (i) the affinity of InOF-1 to toluene and (ii) the unexpected CO_2_ capture enhancement of toluene@InOF-1, powerful periodical DFT and QTAIM calculations were carried out. DFT results shows that the toluene molecule is placed over one of aromatic rings of InOF-1 in a parallel displaced structure,^37^*i.e.*, both molecular rings are nearly parallel but their carbon atoms are not aligned in the perpendicular direction to the planes (see Fig. 4), with an average inter-planar distance of 3.17 Å. This bond length is an expected value for π–π stacking interactions. As it has been previously demonstrated, this type of interactions have a large dispersive component.^38,39^

**Fig. 5:**
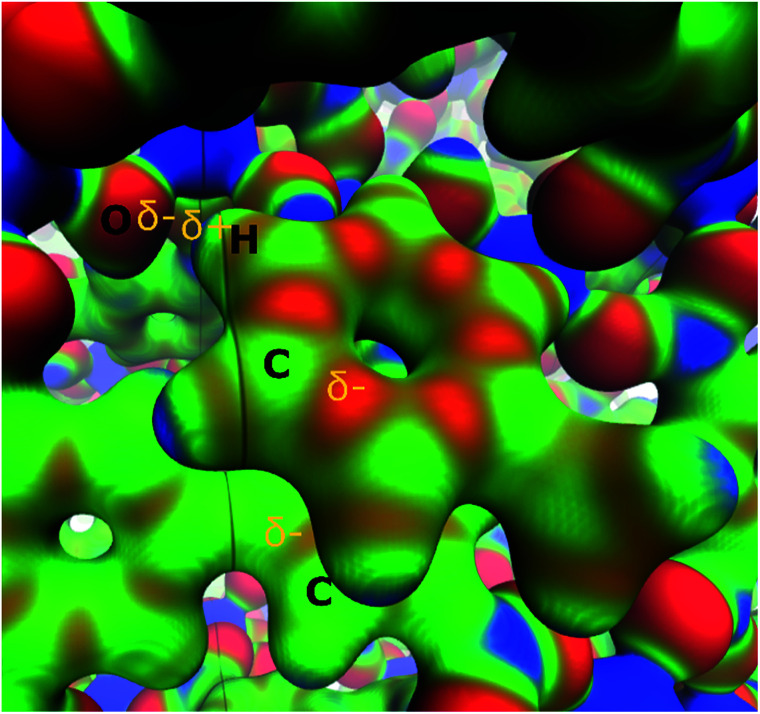
Electrostatic potential of the parallel displaced structure of the toluene molecule adsorbed in InOF-1 over a *ρ* = 0.03 a.u. isosurface (blue 0.01 and red 0.10 a.u., respectively). Partial charges are depicted in yellow. This figure was created with the VMD program.^43^

Nevertheless, although the electrostatic energetic contribution performs a minor role, it has a strong influence on the orientation of the molecules. As can be seen in the electrostatic potential map (MEP) in Fig. 5, the parallel displaced configuration avoids contact between the negative regions (red colour) that are found in the carbon rings of toluene and the biphenyl moiety of InOF-1. Additionally, the MEP also suggests the presence of weak C–H⋯O hydrogen bonds, that can be induced by the contact between the positive areas around the hydrogen atom of toluene (blue colour) with the negative of the oxygen atom (red colour) of InOF-1. Another parallel structure was found with an interplanar separation of 3.339 Å (see Fig. S4, ESI†), but it was not considered in this discussion since it is less stable by 8.1 kcal mol^−1^ and we focus our analysis in the most stable one. There is another geometric arrangement common in non-polar aromatic dimers, where both rings are placed in perpendicular planes^42^ (T-shaped structure). Notwithstanding, this structure was not considered because the toluene molecule could easily be displaced by a gas flux (*e.g.*, CO_2_) since a larger contact area of the molecule (toluene) would be exposed and therefore, pushed out the pores of InOF-1.

**Fig. 6:**
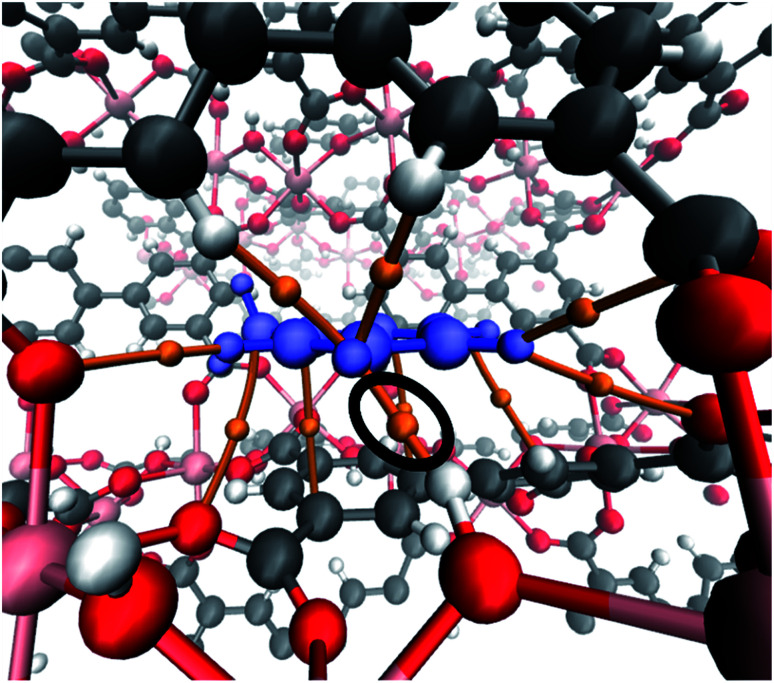
Bond critical points and bond paths (orange points and tubes, respectively) denoting the intermolecular interactions between toluene (blue) and InOF-1. The H⋯H interaction is circled in black colour. This figure was created with the VMD program.^43^

A deeper understanding of the intermolecular interactions responsible for the toluene adsorption in InOF-1 can be acquired by the analysis of the electron density, *ρ*(*r*), and its derivatives. According to the quantum theory of atoms in molecules (QTAIM), the appearance of bond critical points (BCP) and bond paths (BP) indicates the existence of covalent, as well as non-covalent interactions associated to specific atom–atom contacts. In Fig. 6, the BCP and BP corresponding to intermolecular interactions between toluene and InOF-1 are depicted as orange points and tubes, respectively. In agreement with the previous analysis of the MEP, several type of non-covalent interactions where found: C⋯C, that are related to π-π stacking, C–H⋯O, which confirms the presence of this sort of weak hydrogen bond, and the controversial H⋯H contacts, whose nature and consequences are a matter of debate.^44,45^ The value of the electron density at the BCP, *ρ*_B_, is proportional to the interaction strength. From Table S1,† it is noted that the *ρ*_B_ values are around 10^−3^ a.u., which is an order of magnitude lower than those of strong intermolecular interactions such as an O–H⋯H hydrogen bond. Interestingly, an exception was found for a H⋯H contact (circled in black in Fig. 6) between one hydrogen atom of the aromatic ring of toluene and the hydrogen atom of the μ_2_-OH functional group of InOF-1 (*ρ*_B_ = 0.015 a.u.) at 1.812 Å. Even though π–π and C–H⋯O interactions were also found in the structure provided in the ESI (see Fig. S4, ESI†), we propose that the H⋯H contacts are partially responsible for the energy difference of 8.1 kcal mol^−1^. This outcome highlights a new feature of this MOF material; the μ_2_-OH functional group of InOF-1 can contribute to the adsorption of non-polar molecules, albeit in a lower degree in comparison with effect it has in the interaction of polar molecules.^24,27^

**Fig. 7:**
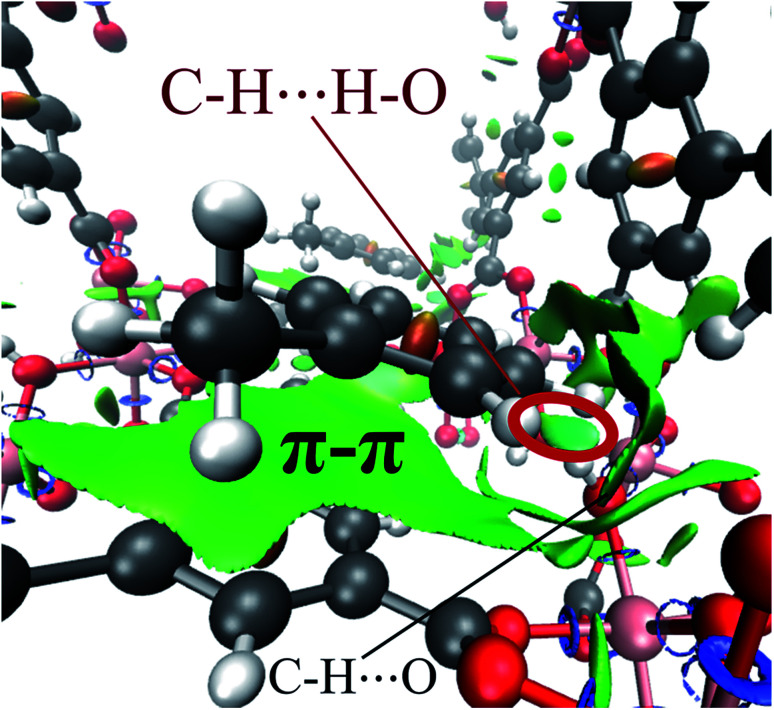
NCI isosurface of the toluene molecule adsorbed in InOF-1 drawn at 0.03 a.u. The main intermolecular interactions are designated. This figure was created with the VMD program.^43^ Some examples of these non-conventional hydrogen bonds can be found in ref. 53.

Finally, by means of the NCI analysis it was possible to elucidate the nature of the present interactions. From the NCI isosurface (see Fig. 7), the existence of π–π stacking is confirmed by the green surfaces that indicates the presence of van der Waals forces between toluene and InOF-1.^33^ The C–H⋯O interactions are visualised by green flat-localised shapes (see Fig. 7) that are characteristic of very weak hydrogen bonds.^41^ The H⋯H contacts also exhibit green extended surfaces attributed to dispersive interactions, except for the one formed between toluene and μ_2_-OH (circled in red in Fig. 7). This contact displays a teal colour disc-shaped region that is typical of medium-to-strong localised interactions.^33,46^ This result corroborates and supports the previous conclusion drawn from the QTAIM analysis.

**Fig. 8:**
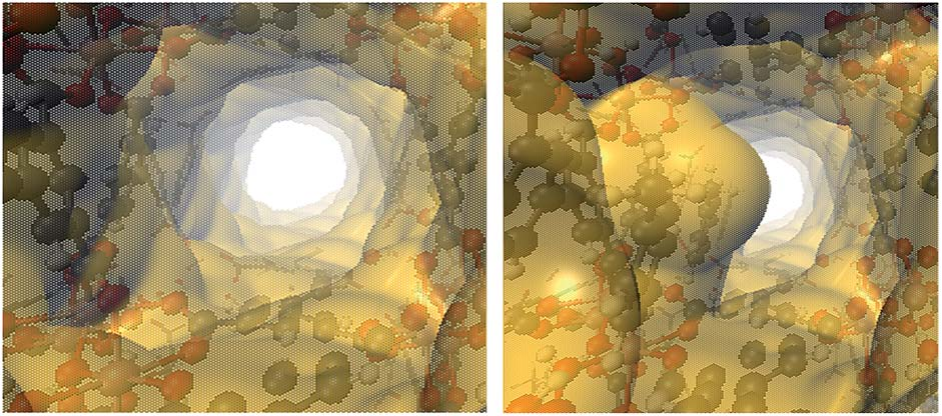
Electron density isosurfaces (*ρ* = 0.0003 a.u.), denoting the void channels of the pristine (left) and toluene-functionalized InOF-1 (right). This figure was created with VMD.^43^

The influence of the functionalisation of InOF-1 with toluene in the CO_2_ capture can be rationalised in two ways: a “bottleneck”^17,24,25,28^ effect, and simultaneous toluene⋯CO_2_ and InOF-1⋯CO_2_ interactions. The former implies a reduction in the pore size, caused by the presence of the adsorbed toluene molecules, that forces the CO_2_ molecules to slow down, enhancing in this way its adsorption. Electron density isosurfaces (*ρ* = 0.0003 a.u.) of the pristine and toluene-functionalised InOF-1, which represent the void channels of these systems,^47^ are depicted in Fig. 8. For our model, where there is one toluene molecule adsorbed in one pore per unit cell (there are in total two pores in the unit cell), the void volume of the corresponding pore is reduced by 19%. This value is 5% larger than previously reported for InOF-1 functionalized with 2-propanol,^27^ and it is plausibly one of the reasons why the CO_2_ uptake is larger for the toluene-functionalized InOF-1.

**Fig. 9:**
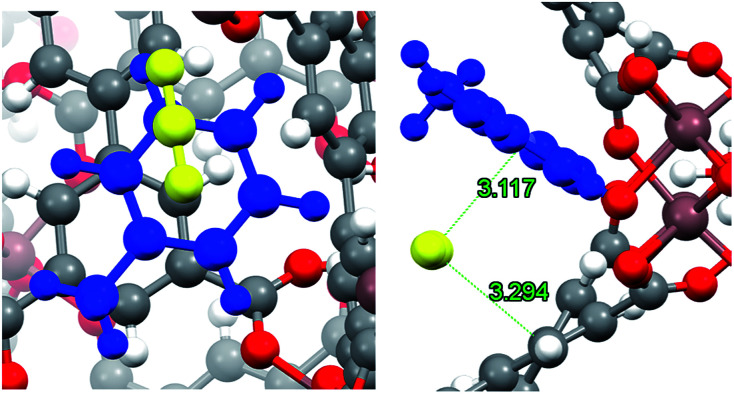
Close up (left) and pore channel (right) views of CO_2_ captured by toluene-functionalized InOF-1. Toluene and CO_2_ molecules are depicted in blue and yellow, respectively. CO_2_⋯toluene and CO_2_⋯InOF-1 intermolecular distances are depicted in green (Å). This figure was created with Mercury.^40,41^

On the other hand, it has been demonstrated that CO_2_ can interact with hydrocarbon aromatic rings,^48–51^ forming complexes where the ring plane and the plane containing the three CO_2_ atoms are nearly parallel. Dispersion forces and attractive electrostatic interactions between the negative π cloud of the aromatic ring and the positive region around the carbon atom of CO_2_ have been held as the guiding causes for this geometric disposition.^48,50^ In Fig. 9, the optimised structure of CO_2_ interacting with the adsorbed toluene molecule is depicted. As expected, the host molecule lies over the toluene ring, with an intermolecular separation of 3.120 Å. It is interesting to note that in this geometric arrangement CO_2_ also interacts with one aromatic ring of the biphenyl structure of InOF-1 at a slightly larger intermolecular distance of 3.294 Å (Fig. 9). Thus, there is a synergic effect between toluene and InOF-1 that also contributes to increase the CO_2_ capture.

### DRIFT spectroscopy

**Fig. 10:**
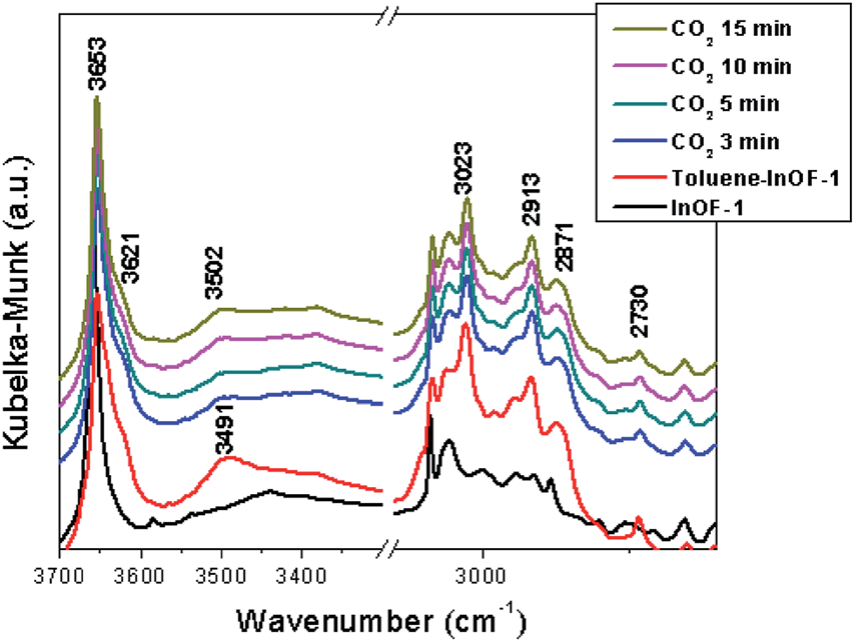
DRIFTs spectra collected at different CO_2_ adsorption times over toluene-functionalized InOF-1 at 30 °C, in the region between 3600 and 2600 cm^−1^.

**Fig. 11:**
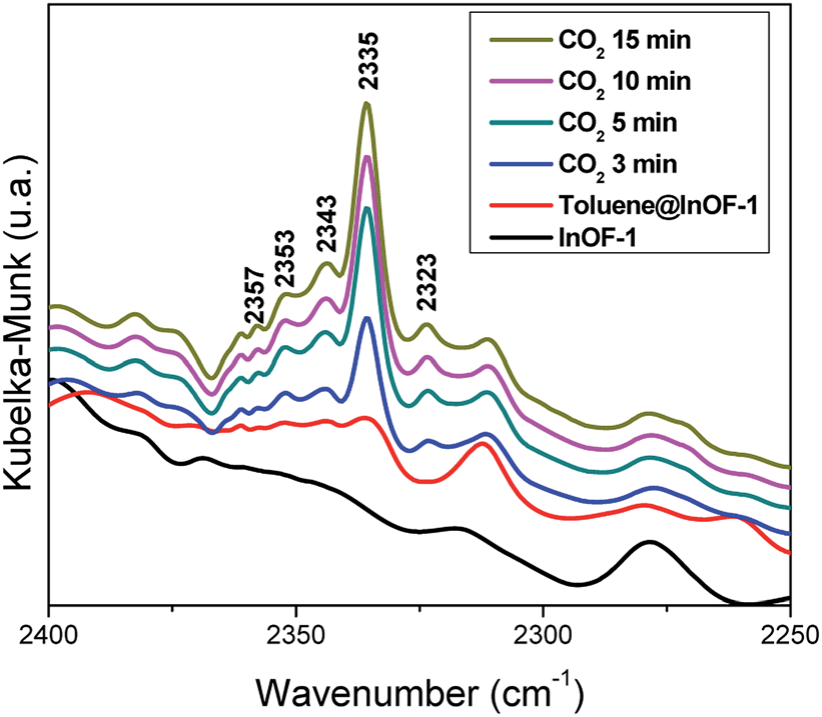
DRIFTs spectra collected at different CO_2_ adsorption times over toluene-functionalized InOF-1 at 30 °C, in the region between 2400 and 2250 cm^−1^.

In order to deeply understand the interactions between toluene-functionalised InOF-1 (toluene@InOF-1) and CO_2_, and to provide experimental support for the interactions proposed from the computational simulations, DRIFTs *in situ* experiments were carried out. Fig. 10 shows the spectra of InOF-1 (as a reference), toluene@InOF-1 and the CO_2_ capture at different times. The presence of the bands at 3023, 2913, 2871 and 2730 cm^−1^ (characteristic of the C–H stretching vibrations of the aromatic ring and methyl group for toluene) observed for toluene@InOF-1 (red spectrum) and absence of these on the activated InOF-1 (black spectrum), corroborates the adsorption of toluene inside the pores of InOF-1. In addition, a peak at 3491 cm^−1^ can be associated to the H⋯H interaction predicted by the computational studies since, as we showed in a previous work,^52^ strong interactions with the μ_2_-OH group of InOF-1 lead to large displacements of more than 200 cm^−1^. Thus, when CO_2_ is captured, the band at 3491 cm^−1^ is shifted to lower energies (3502 cm^−1^), corroborating the interaction of CO_2_ with toluene. Additionally, the CO_2_ region of the DRIFTs spectra is observed in Fig. 11. A vibration band centred at 2335 cm^−1^ is observed. This band is characteristic of the asymmetric stretching modes of the CO_2_ molecule and increases as a function of time. As was previously described, this band does not change significatively, even with strong hydrogen bond formation.^52^ The band at 2323 cm^−1^ can be assigned to the dipole–dipole coupling between adsorbed CO_2_ molecules.^52^ Finally, peaks observed at 2343, 2353, 2357 and 2382 cm^−1^ (see Fig. 11) were assigned to the interactions between CO_2_ and the aromatic rings of toluene and InOF-1, which were suggested by the computational studies. We are know corroborating these findings in studies with other small aromatic systems, such as benzene, and its effect on the adsorption of other substances like SO_2_, which will be published in future works.

These computational analyses and DRIFTs experiments provided us a better understanding on the confinement of small amounts of toluene within InOF-1 (intermolecular interactions), and evidenced a partial obstruction of the pores (similar to other polar molecules like H_2_O, EtOH, MeOH and DMF) which provided a “bottleneck effect” and a synergic effect between toluene and InOF-1, as we previously reported,^17,24,25,28^ to enhance the CO_2_ capture in InOF-1.

## Conclusions

The adsorption properties of toluene in InOF-1 were investigated for the first time, to the best of our knowledge. Rapid adsorption of toluene and hysteresis at 298 and 308 K demonstrated a high affinity towards InOF-1. The affinity of toluene in InOF-1 was quantified experimentally by evaluating the isosteric heat of adsorption (Δ*H*_ads_ = −46.81 kJ mol^−1^). The isothermal CO_2_ dynamic-capture experiments on toluene@InOF-1 demonstrated a CO_2_ capture of 7.28 wt%, which corresponds to an improvement of 1.38 times compared to fully activated InOF-1.

Theoretical results indicate that the toluene molecule is adsorbed through non-localised π–π interactions formed between the toluene molecule and the aromatic ring of InOF-1, which have mainly a dispersive origin, although electrostatic effects control the orientation. Moreover, some specific interactions also contribute to the adsorption process, which are weak C–H⋯O hydrogen bonds and, notably, the H⋯H contact between one hydrogen atom of the aromatic ring of toluene and the μ_2_-OH functional group of InOF-1, which were corroborated by DRIFTs *in situ* experiments. This last result indicates that this functional group of InOF-1 can participate not only in the adsorption process of polar molecules capable of forming hydrogen bonds, but also can take part in the surface assimilation of non-polar molecules such as toluene. This final finding provided the explanation to the experimental CO_2_ capture enhancement (toluene@InOF-1) by the creation of a “bottleneck effect” within InOF-1 and a synergic interaction between the aromatic ring of toluene with the aromatic ligands of InOF-1 and CO_2_.

## Conflicts of interest

There are no conflicts to declare.

## Supplementary Material

RA-009-C9RA05991A-s001
