# Supplementary material for: Confined toluene within InOF-1: CO2 capture enhancement
Source: RSC Adv. 2019 Oct 15;9(56):32864–72. doi: 10.1039/c9ra05991a (PMC9073166; doi:10.1039/c9ra05991a)
Supplement: RA-009-C9RA05991A-s001 [file RA-009-C9RA05991A-s001.pdf]

Electronic Supplementary Material (ESI) for RSC Advances.
This journal is © The Royal Society of Chemistry 2019

Electronic Supporting Information

Confined toluene within InOF-1: CO_2 capture enhancement

L. Pamela Garrido-Olvera,[a] Jonathan E. Sanchez-Bautista,[a] Daniel Alvarado-Alvarado,[a] Bruno Landeros-Rivera,[b] J. Raziel Álvarez,[a] Rubicelia Vargas,*[b] Eduardo González-Zamora,[b] Jorge Balmaseda,[c] Hugo A. Lara-García,[d] Ana Martínez,[c] and Ilich A. Ibarra*[a]*

[a] Laboratorio de Fisicoquímica y Reactividad de Superficies (LaFReS), Instituto de Investigaciones en Materiales, Universidad Nacional Autónoma de México, Circuito Exterior s/n, CU, Del. Coyoacán, 04510 Ciudad de México, México.

[b] Departamento de Química, Universidad Autónoma Metropolitana-Iztapalapa, San Rafael Atlixco 186, Col. Vicentina, Iztapalapa, C. P. 09340 Ciudad de México, México.

[c] Instituto de Investigaciones en Materiales, Universidad Nacional Autónoma de México, Circuito Exterior s/n, CU, Coyoacán, 04510, Ciudad de México, México.

[d] Instituto de Física, Universidad Nacional Autónoma de México, Circuito de la Investigación científica s/n, CU, Del. Coyoacán, 04510, Ciudad de México, Mexico.

1. Powder X-ray diffraction patterns of InOF-1

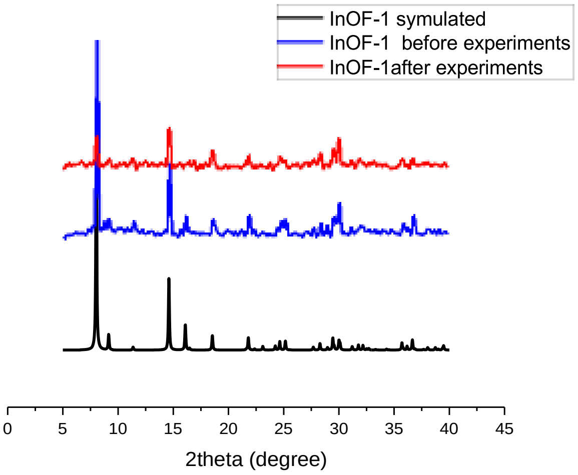

Fig. S1. Powder X-ray diffraction patterns of calculated (black); before experiments, as synthesized (blue); and after-adsorption experiments, without activation (red) InOF-1.

2. Adsorption isotherms for toluene

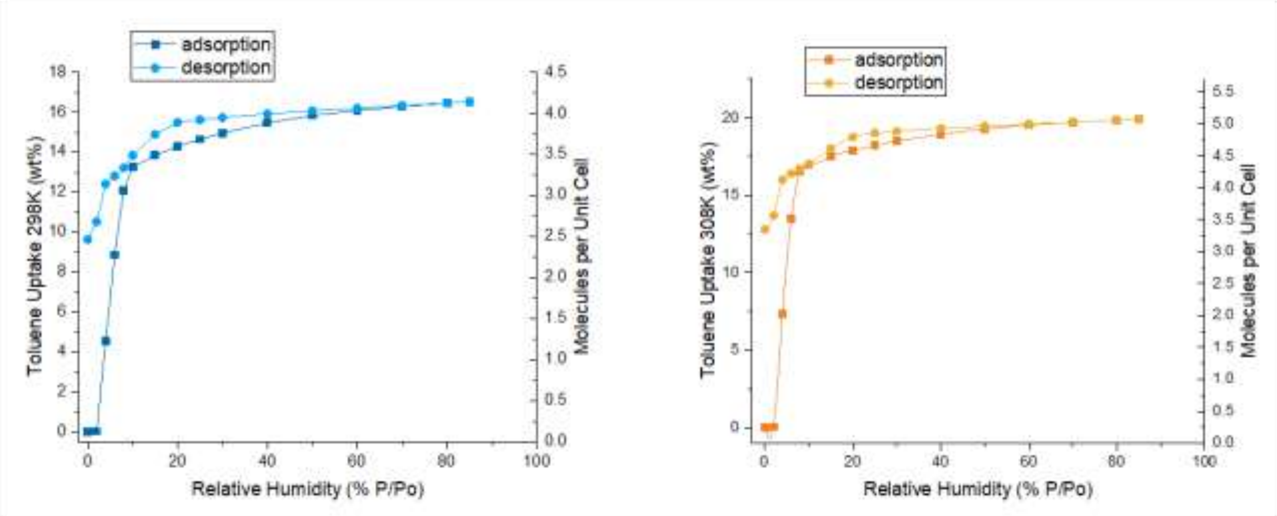

Fig. S2. Toluene adsorption isotherms at 298K (blue) and 308K (orange) for InOF-1 from *from %P/P0= 0 to 85. Squares* show adsorption phase, while circles show desorption phase

3. Derivation of the isosteric enthalpy of adsorption for toluene

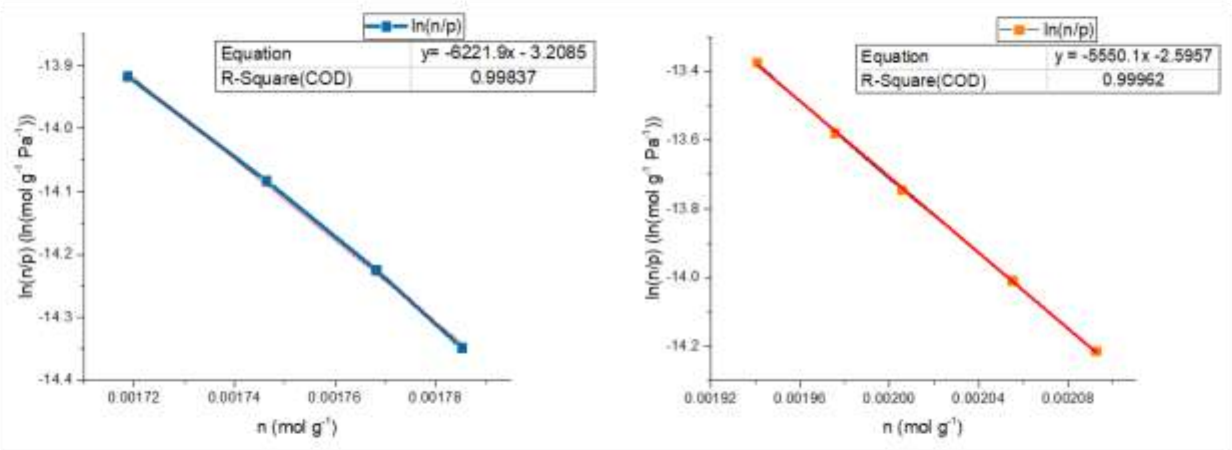

Fig. S3. Viral fitting plot for the adsorption of toluene on InOF-1 at 298 K (left) and 308 K (right)

The isosteric heat of adsorption (ΔH_{ads}) was calculated with the help of toluene adsorption isotherms using the isosteric method, $Q_{"\#}$, by fitting a virial-type equation to both 298 and 308 K toluene adsorption isotherms. The following virial-type equation was used to fit both isotherms:

$$\ln \&\frac{n}{p}) = A_{-} + A_{/}n + A_{0}n^{0} \cdots$$

Where p is the pressure, n is the amount absorbed by the material InOF-1, and A_{-}, $A_{/}$, etc. are the virial coefficients, A_{0} and higher terms can be ignored.

A plot of $\ln 3\frac{4}{5}6$ versus n should give a straight line at low surface coverage. Once obtained the plots, $Q_{"\#}$ was calculated with the help of the Clausius-Clapeyron equation, obtaining a value of 46.81 kJ mol^{-1}:

$$Q_{"\#} = R\,3\frac{8_{9}*8_{;}}{8_{9}<8_{;}}6\; ln\; 3\frac{>_{9}}{>_{;}}6$$

4. Theoretical calculations

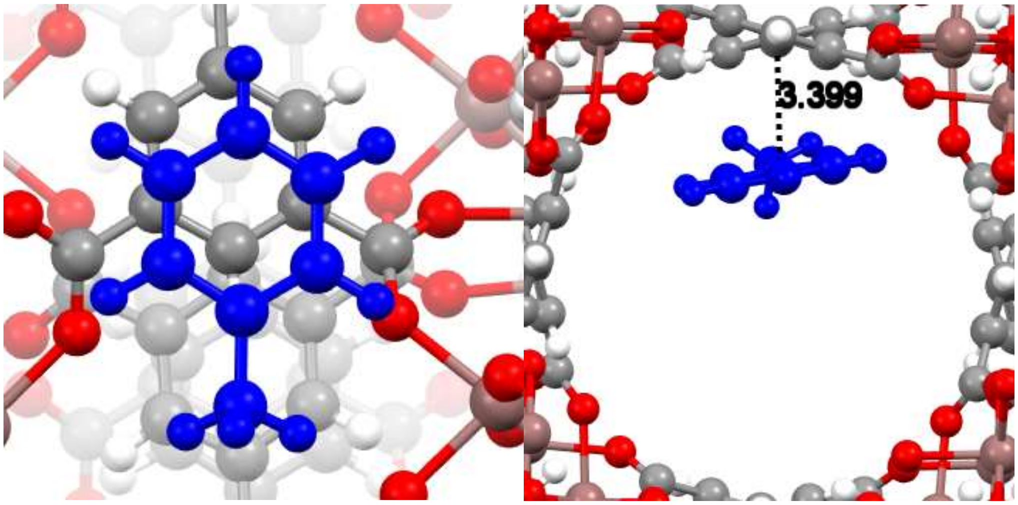

Fig. S4. Pore channel (*top*) and close up (*bottom*) views of an additional optimized structure of the toluene molecule (shown in blue) adsorbed within InOF-1 in a parallel displaced geometry. Pink, red, grey and white colors represent indium, oxygen, carbon and hydrogen atoms, correspondingly. Intermolecular distances are depicted in black (Å).

Table S1. Electron density ($\rho_{@}$), its laplacian ($\nabla^{0}\rho_{@}$), the kinetic and potential energy densities ratio $|V_{@}/G_{@}|$ and the electron energy density ($H_{@}$) values evaluated at the bond critical points for each contact found in the toluene@InOF-1 system. All values (a.u.) are indicative of non-covalent interactions. The strongest H···H interaction is highlighted in black.

Interaction	$\rho_{@}$	$\nabla^{0}\rho_{@}$	$\lvert V_{@}/G_{@}\rvert$	$H_{@}$
C···C	0.0075	0.0271	0.7	0.0014
C···C	0.0076	0.0271	0.7	0.0014
C···C	0.0076	0.0285	0.7	0.0015
C···O	0.0080	0.0286	0.7	0.0015
H···H	0.0089	0.0294	0.8	0.0013
H···H	0.0080	0.0278	0.7	0.0014
**H···H**	**0.0152**	**0.0491**	**0.9**	**0.0014**
C-H···O	0.0083	0.0297	0.7	0.0014
C-H···O	0.0071	0.0241	0.7	0.0014
C-H···O	0.0017	0.0064	0.7	0.0015
